# Weakly supervised learning of biomedical information extraction from curated data

**DOI:** 10.1186/s12859-015-0844-1

**Published:** 2016-01-11

**Authors:** Suvir Jain, Kashyap R., Tsung-Ting Kuo, Shitij Bhargava, Gordon Lin, Chun-Nan Hsu

**Affiliations:** Department of Computer Science and Engineering, Jacobs School of Engineering, University of California, San Diego, 9500 Gilman Drive, La Jolla, 92093 USA; Department of Biomedical Informatics, School of Medicine, University of California, San Diego, 9500 Gilman Drive, La Jolla, 92093 USA

**Keywords:** Biomedical text mining, Natural language processing, Information extraction, Database curation, Machine learning

## Abstract

**Background:**

Numerous publicly available biomedical databases derive data by curating from literatures. The curated data can be useful as training examples for information extraction, but curated data usually lack the exact mentions and their locations in the text required for supervised machine learning. This paper describes a general approach to information extraction using curated data as training examples. The idea is to formulate the problem as cost-sensitive learning from noisy labels, where the cost is estimated by a committee of weak classifiers that consider both curated data and the text.

**Results:**

We test the idea on two information extraction tasks of Genome-Wide Association Studies (GWAS). The first task is to extract target phenotypes (diseases or traits) of a study and the second is to extract ethnicity backgrounds of study subjects for different stages (initial or replication). Experimental results show that our approach can achieve 87 % of Precision-at-2 (P@2) for disease/trait extraction, and 0.83 of F1-Score for stage-ethnicity extraction, both outperforming their cost-insensitive baseline counterparts.

**Conclusions:**

The results show that curated biomedical databases can potentially be reused as training examples to train information extractors without expert annotation or refinement, opening an unprecedented opportunity of using “big data” in biomedical text mining.

**Electronic supplementary material:**

The online version of this article (doi:10.1186/s12859-015-0844-1) contains supplementary material, which is available to authorized users.

## Background

Text mining from the scientific literature has been considered promising for creating and updating structured databases of biomedical knowledge [1] but it often falls short and, currently, manual curation by experts is still the standard practice for this task [2–5]. Some argue that text mining or natural language processing (NLP) becomes unnecessary when researchers report results following a standardized template [6]. Others argue that crowdsourcing may yield better performance than state-of-the-art NLP solutions [7–9]. However, given that scientific publications are still written in free-text and grow geometrically, automatic or semi-automatic approaches may still be necessary for scalable and sustainable data curation [10–13].

### Annotation vs. curation

Machine learning has shown its potential in biomedical NLP and text mining [14–17]. However, supervised statistical learning algorithms require a large number of *annotated* training examples. Annotation and curation are fundamentally different processes. An annotation is a label applied to a span of text. Hence, an annotation appears verbatim in the source text. Annotated databases are rather labor-intensive albeit ideal for text mining applications. On the other hand, curated databases are prepared by domain experts who use a common terminology to describe entities in text. Curated databases require in-depth domain knowledge to produce but the result often requires post-processing before being useful as training examples.

Numerous biomedical databases are available in the public domain. Many of them contain data derived directly from published literature either by curation by teams of experts or submitted by authors or other scientists. A survey estimated that in 2013, a total of 290 papers on biomedical databases that were published that also provided open URL links to access the data. Among these 290 databases, 77.59 % of them collected data from the literature and contained citations as supportive information [18]. However, these data cannot be readily used as training examples because the curated data rarely provide any information of where and how the data were derived from the text.

### Catalog of GWAS

Consider the Catalog of Genome Wide Association Studies (GWAS) [19, 20], an online database developed by the National Human Genome Research Institute (NHGRI) by a curation team of experts. On a weekly basis, epidemiologists from NHGRI and more recently from the European Bioinformatics Institute (EBI) manually curate study-level fields of information from published GWAS and add them to the catalog. As of May 21, 2015, the Catalog of GWAS has been inserted with approximately 29,000 entries extracted from nearly 2200 distinct articles.

**Fig. 1:**
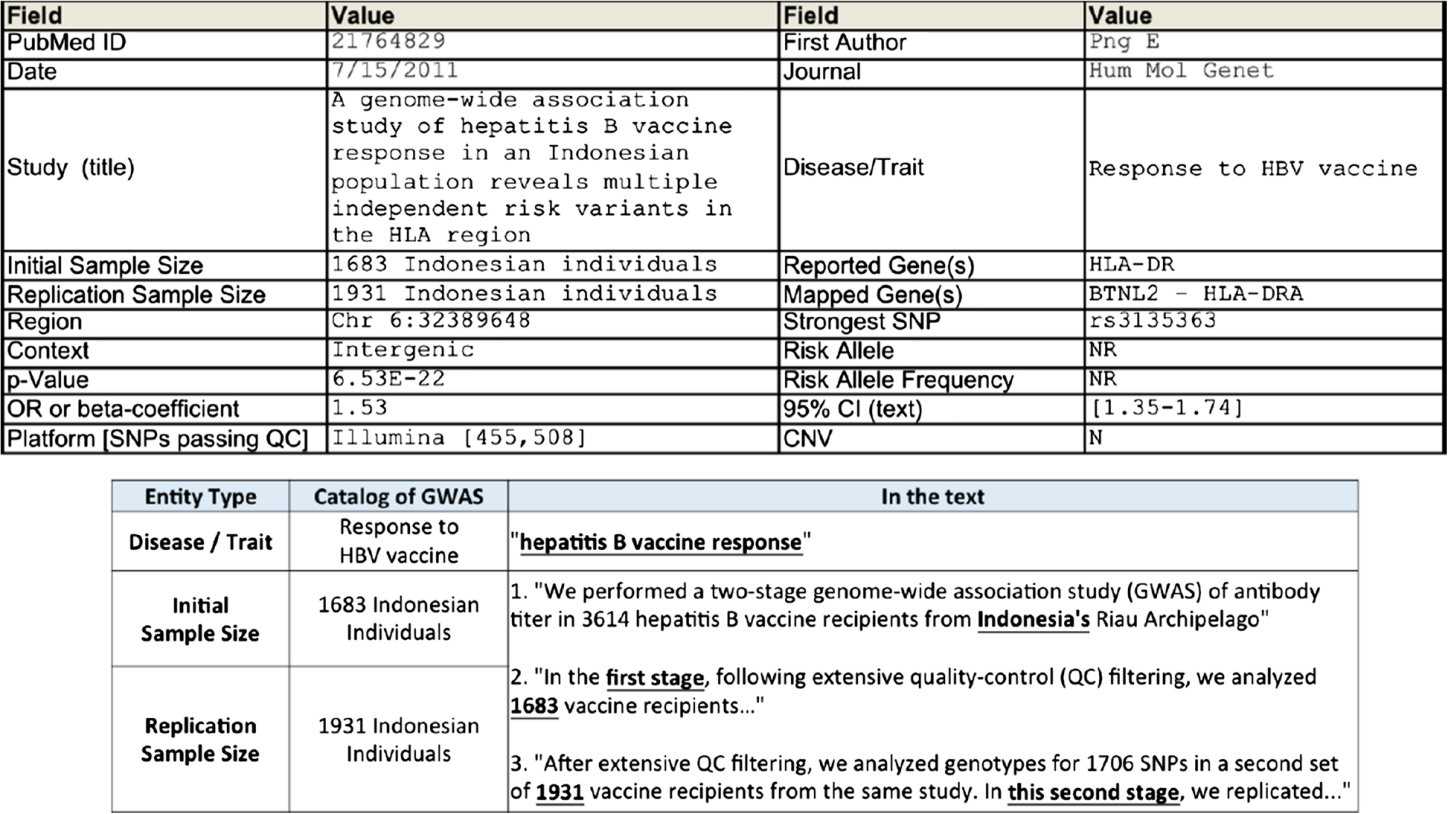
An examplar entry in the Catalog of GWAS and its currated data. Example of an entry in the Catalog of GWAS (*upper panel*) and after matching to the curated data in the text of the source paper [54] (*lower panel*)

Figure 1 (upper panel) shows an example entry in the Catalog of GWAS. Each entry represents an observed association reported in an article, specifying that an association between a genetic variant, given in the data field Strongest SNP, and a phenotype, given in Disease/Trait, was observed from this study from an initial stage sample, given in Initial Sample Size. The entry also specifies that the observation was validated with a replication sample, given in Replication Sample Size. Other data fields include information of where the genetic variant resides in the genome and statistical strength of the observation.

Figure 1 (lower panel) shows the matching result of the entry in the Catalog of GWAS with the actual passages in the text of the article for three data fields. This example illustrates why curated data can be both useful and not useful as training examples. They are useful because matching the data to the text will create training examples. They are not useful because the matching is not trivial. As shown in Fig. 1, matching between the data and text requires background knowledge. In fact, curated data rarely provide verbatim copies of what is mentioned in the source article. For the purposes of easy searching, categorization, summarization, and data integration, curators usually adopt to a standardized terminology different from the text. Also, human curated data inevitably contain typos and inconsistencies in following standards. Even when an exact match with curated data is found, the passage might be about a review of previous results but not the correct location from which the data should be extracted. To sum up, curated data are useful but imperfect as training examples.

### Learning from curated data

This paper describes a general approach to using curated data from existing biomedical databases as training examples for information extraction from full-text research articles. Our approach is based on results of research in agnostic learning from data with noisy labels [21–27]. Among the results that fit our need here is *importance reweighting* [21], where training examples are weighted according to the reliability of their labels, which, however, is unknown in practice and must be estimated. Our solution is to employ a committee of weak classifiers that match candidate passages in the input text with curated data and then apply the EM algorithm to estimate the reliability of the labels. Then we can use a *cost-sensitive* learning algorithm that learns from training examples weighted by their misclassification costs derived according to the estimated reliability to develop accurate information extractors.

The applicability of this approach is not limited to the Catalog of GWAS. A large number of biomedical databases are available in the public domain, and many contain data derived directly from published literature either through manual curation by teams of experts or structured information submitted by authors or researchers. We intend for our approach to be generalizable across these databases as well.

The problem of learning from imperfect training examples is closely related to *entity normalization*, where the training examples available are normalized entity names rather than annotations in the text. For example, the gene normalization task in the BioCreative II and III initiative [28–32] aiming at extracting standardized gene IDs mentioned in an input article provided only the standardized gene IDs for each article as the training examples. The problem is also related to learning from data with noisy labels and learning from crowds [23, 33–35], where crowd inputs are considered noisy.

### The test tasks

We implement the approach and apply it to two problems of information extraction from the full-text research reports of GWAS. Task 1 is to identify target phenotypes (disease/trait) examined in a GWAS study. This task is different from well-studied disease mention tagging and normalization [36–38] in that not all mentions but only the study targets need to be identified and that GWAS targets include not only diseases but a wide range of traits like eye color, response to ximelagatran treatment, sleeping habits, reading and spelling ability, education attainment, political ideology, etc. Usually, a GWAS targets a single phenotype, but a study may examine more than one phenotype. This is often the case when the study target is a complex disease, such as obesity, for which scientists may seek genetic associations with related traits such as body mass index, waist-height ratio, waist circumference, etc., in addition to direct association with obesity.

**Fig. 2:**
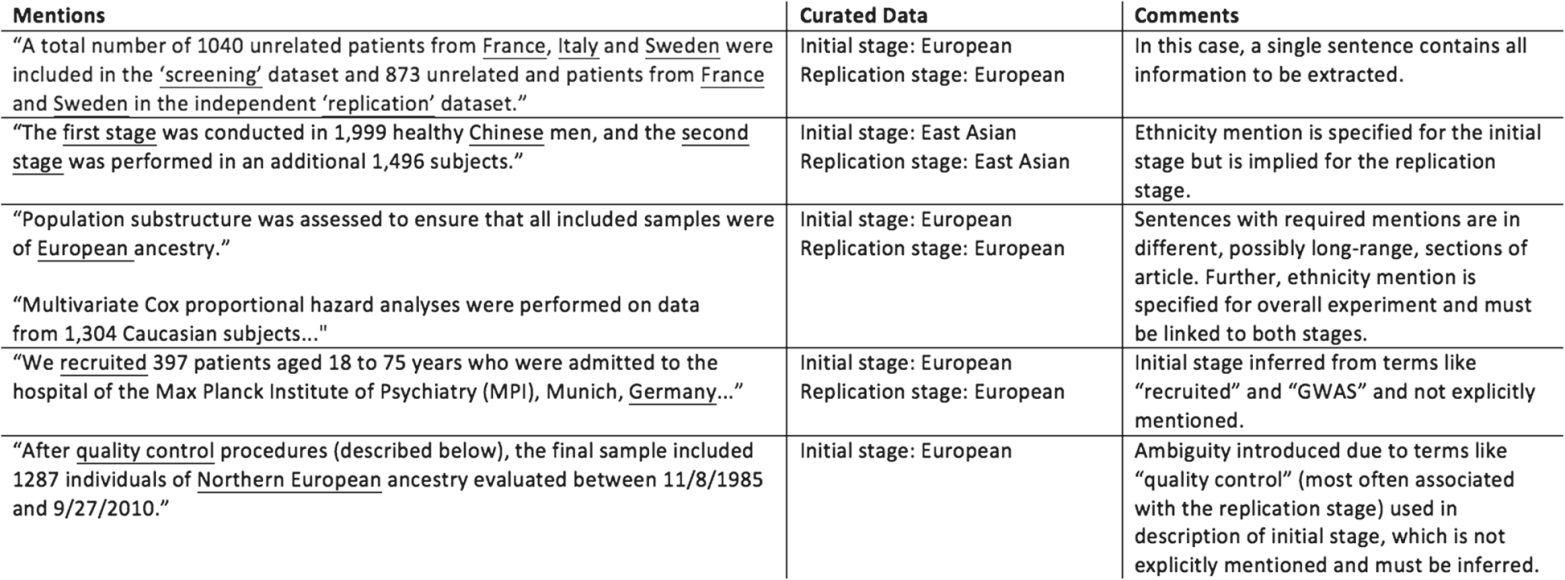
Example sentences describing sample ethnicity groups and stages. Example of sentences in free text from which the system extracts study targets

Task 2 is to extract stage (“initial” or “replication”) and ethnic group of the study samples. A GWAS involves study samples drawn from one or more ethnicity groups. Task 2 is concerned with the problem of extracting these ethnicity groups that the experiment pertains to. There are conventionally two *stages* in the study: initial and replication, and each of these can be associated with several distinct sample populations. However, many articles may not specified all information clearly in the text. Figure 2 illustrates some examples for this task. The first row gives an ideal case for information extraction, where both stages and their corresponding ethnicity information are specified in a single sentence, though it is still nontrivial to normalize terms in the sentence to a common terminology (i.e., mapping “screening” to “initial” and “France”, “Italy”, and “Sweden” to “European”, the top-level ethnicity group, according to the curation guideline [39]). Figure 2 also contains examples of sentences where an ethnicity mention is not explicitly linked to either stage, or is specified for one stage and must be inferred for the other. This can be exacerbated when these mentions are far apart in a full text.

Task 1 is an entity recognition problem while Task 2 requires jointly extracting different attribute types and identifying linkage between them. For both tasks we develop information extractors by applying the same general approach to learning from the curated data in the Catalog of GWAS as the training examples. We are able to achieve 87 % of extraction in Precision-at-2 (P@2) for the Task 1 and 0.83 in F1-Score for the Task 2. Both outperform their baseline, cost-insensitive counterparts. The remainder of this paper describes the method and the results in details.

## Methods

We start by describing our general approach and then follow by presenting the implementation of the general approach for the two test tasks.

### The approach

**Fig. 3:**
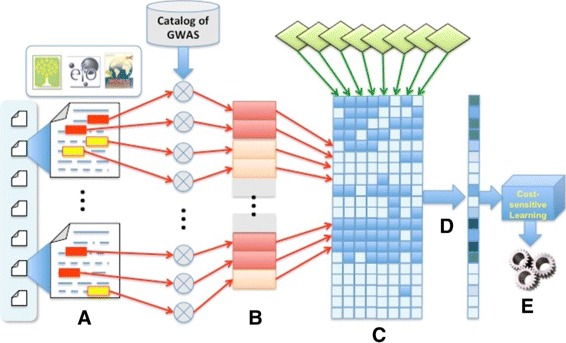
System architecture. Our proposed system architecture summarizing the steps (**a**-**e**) in the machine learning training process

Figure 3 shows the five components and the workflow of the whole learning approach. The input is a large corpus of research articles for training. For each article, **Step (A)** identifies the passages that may contain the information to be extracted in the text. The identification of passages should be inclusive in the sense that any candidate passages will be extracted and no passage is missed.

**Step (B)** pairs each passage with a piece of matched curated data and creates a feature vector for the pair as the input to the committee classifiers. For example, we pair passage 2 in Fig. 1 (lower panel) to data item “1683 Indonesian Individuals” from the Catalog of GWAS, because passage 2 is likely from where the data item was derived. Again, the matching should be inclusive to contain all potential pairs.

**Step (C)** then sends the feature vectors to a committee of classifiers (diamonds on top of Fig. 3). Each classifier classifies each pair into positive, if the passage is deemed to contain the information given in the curated data, or negative otherwise. The classifiers can be as “weak” as simple decision rules, like “whether the passage contains a substring that exactly matches the curated data”. Therefore, each committee member classifier provides noisy positive-negative labels of the passages extracted from the text. Combining the classification results of all committee members for all extracted passages creates a a large matrix of yes-or-no votes, where each element (*i,j*) contains the vote from classifier *i* for candidate passage *j*.

**Step (D)** estimates from the matrix the probability that candidate passage *j* is truly positive by a label estimator that applies an Expectation-Maximization (EM) algorithm to compute maximum likelihood estimation of the probabilities, which can then be treated as the weight, or the reliability of a candidate training example. A similar approach was used in the BioCreative III gene normalization task [29] to create a silver standard. The EM algorithm works as follows:

1. **Input**: matrix ***M*** of committee (column)-passages (row), where each element in the matrix is either positive (=1) or negative (=0);
2. Let *p*_*i*_ be the probability that the *i*-th passage should be positive, *e*_*j*_ be the error rate of the *j*-th committee classifier; Let *t*=0;
3. Initialize *e*_*j*_(0)=0 for all *j*;
4. Update for all *i*, $p_{i}(t) = \frac {\sum (1-e_{j}(t-1))\boldsymbol {M}_{\textit {ij}} + k}{J+K}$, where *J* is the number of weak classifiers in the committee, *k*/*K* is the Laplace prior;
5. Update for all *j*, $e_{j}(t) = \frac {\sum p_{i}(t)\boldsymbol {M}_{\textit {ij}} +k'}{I+K'}$, where *I* is the number of the passages, *k*^′^/*K*^′^ is the Laplace prior;
6. *t*=*t*+1 and repeat update steps until convergent;
7. **Output**: $\hat {p_{i}}=p_{i}(t)$ for all *i* and $\hat {e_{j}}=e_{j}(t)$ for all *j* as the final values.

With the estimated probability of each candidate passage, we can assign it a *cost*, and train a cost-sensitive learner [40–42] using the candidate passages as the cost-weighted training examples to learn to select correct passages that contain the desired information as **Step (E)**. The cost that we use here is derived according to Lemma 1 in [21], where the problem of classification with noisy labels is solved by importance reweighting. They show that an error bound can be achieved if the misclassification cost of a training example (*x,y*) is set to *p*(*y*|*x*)/*p*_*ρ*_(*y*|*x*), where *ρ* denotes sampling from a noise-perturbed distribution. Though neither *p*(*y*|*x*) nor *p*_*ρ*_(*y*|*x*) are known, we can approximate *p*(*y*=“+” |*x*) by $\hat {p}$ from the EM algorithm above and *p*_*ρ*_(*y*=“+” |*x*) by $p(\hat {p}(y=$“+” |*x*)>0.5) for a training example estimated as positive and analogously for a negative one. That is, let $y_{i} = \text {round}(\hat {p_{i}})$. If *y*_*i*_=1 then the cost of misclassifying the *i*-th passage as negative is $c_{i} = \frac {\hat {p_{i}}}{\sum _{i} y_{i}/I}$, else $c_{i} = \frac {\hat {p_{i}}}{1-\sum _{i} y_{i}/I}$.

We note that this cost-sensitive classifier may use a completely different set of features to characterize a passage.

After the cost-sensitive learning completes, to extract desired data from a new article, we apply the same **Step (A)** to extract candidate passages and send them to the trained cost-sensitive classifier and extract data from the candidate passages classified as positive.

### Task 1: Identifying target phenotype (disease/trait)

#### Data

The curated GWAS target phenotype data are in the form of a spreadsheet dated May 2014 available for download at [43]. Each row in the spreadsheet contains a column DISEASETRAIT, reporting a phenotype term chosen by curators as the study target of the paper with its ID given in column PUBMEDID. As we discuss earlier, these phenotype terms do not always exactly match what is mentioned in the text. Column EFOTRAIT contains the phenotype term mapped to a concept defined in Experimental Factor Ontology (EFO) [44], the standard terminology of diseases and traits for the Catalog of GWAS. EFO classifies concepts into 17 high-level categories as given in column PARENT. For example, DISEASETRAIT for the paper of PubMed ID 18849991 is “Male-pattern baldness”. The corresponding EFOTRAIT and PARENT are “androgenetic alopecia” and “Other disease”, respectively. These columns constitute the curated data that we intend to use to train our information extractor.

The spreadsheet contains data for 1742 unique PubMed IDs of GWAS articles. Among them, 307 papers have full text available in the NXML format [45] from PubMed Central. NXML provides useful metadata for NLP and text mining such as section headings to distinguish main text from references and other elements but currently only about one third of all PubMed papers have their NXML version available from PubMed Central. For other papers, we collected 965 full texts in PDF. These PDF files were all transcribed into XML using bioPDFX [46], a tool that we built on top of PDFX [47], to prepare PDF papers for biomedical text mining.

For the purpose of evaluation, we manually augmented the data with the corresponding terms that actually appear in the text of the 307 NXML articles to serve as our hold-out gold standard. We kept the data of the 965 PDF papers intact and used the data for training.

The spreadsheet contains a total of 2645 rows for 1742 unique papers. That is, each paper has on average 1.51 target phenotypes. Three hundred thirty three papers (19.11 %) have more than one target phenotypes in the curated data. We therefore measure the performance based on the precision-at-2 (P@2) metric, that is, if either of the top 2 extracted phenotypes match the gold standard, the extraction is considered correct.

#### Implementation

**Step (A): Passage extractor.** In this step, we identify all mentions of any disease or trait using an exact string matching approach, which is based on a dictionary of all diseases and traits from the search menu of the web query interface of the Catalog of GWAS at [48].

After string matching, we extracted 117,384 mentions in the training and 72,914 in the test data. Note that these numbers are the total mentions of any disease or trait in all articles in the data set, because a paper usually contains multiple mentions.

**Step (B): Feature creator.** The following features are generated for each training sample:

- *Token-based features*: Character-level n-grams of the mention.
- *Context-based features*: Word-level and character-level n-grams for up to 10 words before and after the mention.
- *Position-based features*: The location of each mention can be indicated using positional tags (e.g., 〈article-title 〉 and 〈*abstract*〉) in the converted XML papers; therefore, whether a mention is located within positional tags are extracted as binary features. These tags, however, are not always available from the PDF-transcribed XML versions.

For each mention, token-based and context-based features are represented as normalized TF-IDF vectors. Together with position-based features, each mention has approximately 120,000 features.

**Step (C): Committee of classifiers.** To build the committee matrix, we design five rule-based binary classifiers as follows.

- *Title or Abstract*: Whether disease/trait mention occurs in the title or abstract of the paper; this is a simple yet strong indicator of a disease mentioned being the actual target of the paper.
- *Exact match*: Whether disease/trait mention exactly matches the target given by human curator.
- *Sub-string match*: Whether disease/trait mention partially matches the target given by the human curator (e.g., a mention of “Diabetes” would be classified as positive, if the human curator determines the disease as “Type-2 Diabetes”).
- *Synonym*: Whether disease/trait mention is an exact or partial match of a synonym of the target determined by the human curator. The synonyms are collected from UMLS [49]; for a given disease or trait mention, all UMLS concepts that shared the same CONCEPT-ID are considered to be synonymous. To reduce noise, we only keep the synonyms which are in English and are preferred terms (i.e., the IS-PREF flag set to Y in UMLS).
- *Compound token*: Whether the mention has multiple tokens separated by a space or hyphen (e.g., “Parkinson’s disease” would be classified as positive because it consists of compound tokens separated by a space).

It should be noted that although some rule-based classifiers are extremely weak (e.g., compound token), our idea is to show that multiple weak classifiers can actually contribute to a strong committee and make accurate predictions.

**Step (D): Label estimator.** We apply the EM method described previously to label each pair of passage and curated disease or trait with an estimated confidence (i.e., the conditional probability given the pair is positive).

**Step (E): Cost-sensitive learner.** In this step, we utilize the estimated confidence generated by the Label Estimator to assign the cost to train a cost-sensitive variant of Support Vector Machine (SVM).

**Post-processing.** Each paper may contains multiple mentions of various disease and traits. For example, a paper may contain 10 mentions of “Diabetes”, and 30 mentions of “Hypertension”. However, our cost-sensitive classifier may predict only part of them to be positive. This is reasonable because even though two sentences mention the same phenotype, it is not always the case that both are stating that the phenotype is the study target. We consider the following two scores for the post-processing, inspired by TF and IDF, respectively:

1. $P_{\textit {TF}} = \frac {V_{\textit {pi}}}{V_{\textit {pi}}+V_{\textit {ni}}}$, where *V*_*ni*_ is the number of negative votes assigned to the *i*-th candidate.
2. $P_{\textit {IDF}} = \frac {V_{\textit {pi}}}{\sum V_{\textit {pi}}}$, where *V*_*pi*_ is the number of positive votes assigned to the *i*-th candidate.

To combine *P*_*TF*_ and *P*_*IDF*_, we apply two mean computations, namely arithmetic and harmonic, to calculate the final scores and determine our predicted disease/traits. The harmonic mean better represents the mean value of these two metrics. That is because the *P*_*TF*_ and *P*_*IDF*_ values are often quite small and include outlying values. Harmonic mean is a more sensitive measure in such cases.

### Task 2: Identifying stage and ethnicity of study samples

We represent this problem as that of extracting tuples of the form 〈stage, ethnicity 〉 from the free text of a GWAS article, with the entities in the tuple corresponding to stage and ethnicity of the study sample.

#### Data

Again, our articles are selected from the Catalog of GWAS. The data in the form of a spreadsheet is available for download at [48].

We selected articles that satisfy the following criteria:

- *Curated data available*: 2185 PubMed articles were curated with the data available.
- *NXMLs or PDFs available*: We used NXML versions of the articles if they are available through PubMed Central. These versions have high-quality text. Otherwise, we transcribed PDF versions of the remaining articles to text. This leaves 1861 articles.
- *No missing values or “NR”*: The characteristics of the samples are available for whichever stage is mentioned in the article, and the curated data contain no blank entries. Also excluded are those for which curators were unable to find a conclusive ethnicity group for the sample and the entries state “NR” (“not reported”). This leaves 1674 articles.
- *Ethnicity mentions in text*: Terms that correspond to ethnicity groups must be available in text (but not inferred from affiliations of authors, for example).
- *Do not contain errors*: The curated data was found to contain errors in the entries for some articles. Those were excluded.

The final dataset consists of 1311 articles, comprising 2357 〈stage, ethnicity 〉 tuples.

The curated data is normalized to remove spelling errors and inconsistent wording primarily to ensure that there is only one top-level term for a given ethnicity entity. For example, ethnicity group entries in the curated data stating “North African/Middle East” or “Middle East/North African” are both considered to correspond to “Middle East/North African”, with this choice of the eventual top-level entry being made arbitrarily.

#### Implementation

We applied the same pipeline given in Fig. 3 but we employed two committees to extract tuples with two elements: **Step (A)***Passage extraction*: Mentions in the text corresponding to ethnicity entities are tagged and their surrounding passages extracted. These instances are (weakly) labeled according to curated data as positive or negative. **Step (B)***Feature creator*: The ethnicity instances are featurized and made suitable for classification. **Step (C)***Committee of positive/negative classifiers*: A committee of weak learners are exploited to generate noisy labels, for cost-sensitive learner to classify ethnicity instances as positive or negative instances. *Committee of initial/replication classifiers*: Ethnicity instances classified as positive are further classified into the initial and replication experimental stages of the GWAS. For both committees, we perform **Step (D)***Label estimator* using EM algorithm, followed by **Step (E)***Cost-sensitive learner* to predict the ethnicity and stage of the mentions. *Post-processing*: Instances of *ethnicity* classified into a particular *stage* are grouped as 〈stage, ethnicity 〉 and duplicates removed. The performance of this method is evaluated upon this final set of results.

These steps are described in detail below.

**Step (A): Passage extractor.** Mentions in text are generally not exact string matches (or even exact synonyms) of ethnicity groups, necessitating a dictionary mapping of mentions in text (e.g., “German”) to the top-level ethnicity entity (e.g., “European”). Mentions in the text that correspond to a top-level ethnicity entity are mapped to their corresponding entities and tagged with the help of a constructed dictionary as described below, followed by passage extraction. Mentions corresponding to a stage are not tagged.

We construct the dictionary of ethnicity mappings as follows. A multitude of terms can refer to the ethnicity of an individual, including the country of origin (e.g., “Germany”), the specific ethnicity group (e.g., “European”), an adjectival for the country (e.g., “German”), a demonym for the country (e.g., “Germans”), and similar sets of terms for cities and other regions. We handle these terms through the conventions:

- *Country/region name*: Not every mention of a region, say, a country, maps to a specific ethnicity term. The NHGRI curation guideline [39] stating that a given set of individuals belong to an ethnicity group only if it is directly stated in the study, or if at least 90 % of the population of the region is known to belong to a single ethnicity group, with this knowledge being based on the CIA World Factbook [50].
- *Adjectivals and demonyms*: An extensive list of the adjectivals and demonyms for countries are obtained from Wikipedia and a dictionary is constructed to map the terms to their corresponding countries. These countries are then mapped to the corresponding ethnicity group (or discarded if no mapping exists).

The final dictionary comprises 449 terms that map to 14 top-level ethnicity groups. These terms cover a majority of the mentions in text, and we omit publications that do not contain language that can be matched to this dictionary. A more comprehensive dictionary may include lists of tribes and indigenous peoples of the world.

This dictionary is used to match mentions in text to ethnicity entities through string matching. The tagged instances are extracted along with their corresponding passages, which consist of the 10 words on either side of the entity in the sentence.

For training and testing, these instances are weakly labeled from curated data by checking if, for a given article, the ethnicity group is present in either experimental stage, initial or replication. If so, this is considered a positive instance, and negative otherwise.

**Step (B): Feature creator.** The following types of features are generated for each instance:

- *Token-based features*: A set of binary features each of which turn on for a specific ethnicity entity (e.g., a feature will be 1 for “East Asian”).
- *Context-based features*: These include normalized term frequency-inverse document frequency (TF-IDF) representations of unigrams and bigrams of 10 words in either direction of the ethnicity mention, as long as the words are within the same sentence. The words are stemmed using the Porter stemmer [51].
- *Position-based features*: These include features like section title (also in TF-IDF form), the distance (normalized) of the ethnicity mention from the start of the article, or from the start of the section.
- *Additional features*: These include features that do not fit into the above categories, such as the number of times the ethnicity entity was observed (tagged) in the same article.

This results in sparse feature vectors of approximately 80,000 dimensions. The features are normalized by removing the mean and scaling to unit variance across the values of each feature, or dimension of feature vector. As the feature set is mostly composed of various TF-IDF vectors, truncated Singular Value Decomposition (SVD), or Latent Semantic Analysis, was explored as a feature selection technique to reduce the dimensionality of the feature vectors. However, this did not affect the performance significantly (and in fact degraded performance slightly) and hence the complete feature vectors were retained. The complete list of the features are given in (Additional file 1).

**Step (C): Committee of positive/negative classifiers.** We use the cost-sensitive learning approach described previously to classify instances as positive or negative. The committee members of weak labelers include:

- *Binary classifier*: the results of a Logistic Regression binary classifier trained on the weak labels from curated data.
- *Rule-based classifier*: this classifier predicts a positive example if the features meet any criteria, such as the presence of words that are commonly found in descriptions of a sample (e.g., “stage”, “cohort”). 65 such terms are used in total.
- *Weak labels from curated data*: the labels obtained by exact-matching the ethnicity to the curated data.

The committee matrix obtained from concatenating the outputs of all the members is used to estimate the cost to be assigned to each training instance, as in previous sections. These costs are used to train a cost-sensitive, *L*_2_-regularized, linear support vector machine (SVM) classifier to classify instances as positive or negative instances.

**Committee of initial/replication classifiers.** Training a cost-sensitive classifier to classify positive instances of ethnicity entities into the corresponding stage of a study is performed in a similar fashion to that for the ethnicity instance classifier.

In this case, the positive training instances are now relabeled “initial” or “replication”. The committee members are:

- *Binary classifier*: as above, but trained to distinguish “initial” from “replication” instances.
- *Rule-based classifier:* the rule-based classifier is modified to use the presence of stage-specific words to make its prediction (e.g., “discovery” for the initial stage, or “follow-up” or “second stage” for replication). 8 such terms are used.
- *Weak labels from curated data:* as above, but containing classes “initial” and “replication” instead.

The outputs of the members are used to construct the committee matrix and estimate the cost assigned to each training instance, which is then used to train a cost-sensitive, *L*_2_-regularized, linear SVM to classify the test data into the initial or replication stages with the same set of features.

The output of this step is a classification of each positive *ethnicity* instance into a specific *stage* (initial or replication).

**Post-processing.** Either stage in a GWAS may have multiple ethnicity groups. Hence, the extraction can possibly result in multiple tuples of the form 〈stage, ethnicity 〉 for each study. We compile a list of such tuples for each article, with duplicates being discarded.

## Results and discussion

### Results of task 1: Identifying target phenotypes (disease/trait)

We use precision at 2 (P@2) to measure the performance because a GWAS may examine one or more phenotypes. If either of the top 2 extracted phenotypes match the gold standard, the extraction is considered correct.

We compare our cost-sensitive approach with a cost-insensitive baseline. Also, we attempted the following additional alternatives to improve the cost-sensitive learner:

- *BIOADI*: Since in many articles, the target phenotype only appears once in its full form and all following mentions are in its abbreviation, we identify and normalize the abbreviations in the input text using the BIOADI system [52] to pre-process the text in an attempt to improve the performance.
- *Conditional Random Field (CRF)*: In order to deal with new diseases and traits that do not appear in our training dictionary, we also tried to apply CRF in the Passage Extractor step. The design of the features for the CRF is based on the method described in [53]; we use a mixture of general linguistic, orthographic, contextual, syntactic dependency, and dictionary lookup features. By using this CRF model, we discover 59,648 mentions in test data, which is, however, less than the number of mentions using dictionary matching.

We train all systems with the data of 965 articles and test the trained systems with the hold-out data of 307 articles. Table 1 shows the performance results of these systems. The cost-sensitive learner outperforms the cost insensitive learner and the harmonic averaging outperforms arithmetic averaging. However, the alternatives to improve passage extraction (BIOADI and CRF) fail to improve the result of the cost-sensitive learner.

**Table 1:** Precision-at-2 (P@2) of identifying target disease/trait mention of a GWAS study

Method	Arithmetic	Harmonic
Cost-insensitive	68.65 %	79.62 %
Cost-sensitive	78.05 %	87.46 %
BIOADI+Cost-sensitive	75.57 %	87.29 %
CRF+Cost-sensitive	65.79 %	75.24 %

We experiment with more than 2 extractions as well. If we consider top five extractions, the accuracy of at least one of them being correct is higher than 93 %. If we consider a single extraction, the accuracy drops by a few percentage points but stays above 82 %.

### Discussion of the results of task 1

The experimental results show that learning from curated data is feasible to accomplish a P@2 up to 87 % for the task of extracting study target and that the cost-sensitive learning approach outperforms the cost-insensitive baselines.

We analyze the errors and summarize that many errors are from pharmacogenomic studies, which examine phenotypes such as “Response to antipsychotics” as given in the curated data. Antipsychotics are a class of psychiatric medication instead of a specific medication, while the disease targets of the medication, such as “schizophrenia”, “major depressive disorder”, etc., may present stronger signal as the study target than the response of a medication for an information extractor. Studies of complex diseases also pose a main challenge because a complex disease may have many associated measurable traits.

### Results of task 2: Identifying stage and ethnicity of study samples

We evaluate the performance by comparing them with the 〈stage, ethnicity 〉 tuples known to correspond to each GWAS article. Further, we also compare the results with the following alternative approaches. The evaluation methodology and metrics are described below.

1. *Baseline*: All ethnicity instances tagged by the dictionary in an article are assigned to both experimental stages, and the results measured.
2. *Cost-insensitive classification*: the framework described above is used in a cost-insensitive fashion by excluding the committees and directly training the classifiers on the curated labels. This provides a candidate for comparison to the cost-sensitive approach for evaluating the performance of cost-based learning.
3. *Cost-sensitive classification*: the framework described above, including committee classification, is used.

In each of the methods (excluding the baseline), five-fold article-based cross validation (5-fold CV) is performed. The articles in the dataset are randomly shuffled, and each fold of the 5-fold CV utilizes all 〈stage, ethnicity 〉 tuples belonging to 80 *%* of the articles in the dataset as training data, and the tuples in the remaining 20 *%* of articles as test data.

The results from each fold are then collected to obtain 〈stage, ethnicity 〉 tuples for all the articles in the dataset. These results are compared against the curated data and the F1 score calculated in the standard way:

- If a 〈stage, ethnicity 〉 tuple in the result for a specific article is present in curated data for that article, it is considered a true positive (TP); otherwise, it is considered a false positive (FP).
- If a 〈stage, ethnicity 〉 tuple in the curated data for a specific article does not have a counterpart in the extracted results, it is considered a false negative (FN).

Using this, we calculate the precision, recall and the Macro F1 score for each method on 1,311 articles comprising 2,357 〈stage, ethnicity 〉 tuples from approximately 35,000 mentions of ethnicity-related terms. The resultant values are tabulated in Table 2.

**Table 2:** Performance of stage-ethnicity extraction (micro average)

Method	Precision	Recall	F1 Score
Baseline	0.4898	1.0000	0.6576
Cost-insensitive	0.6965	0.7077	0.7020
Cost-sensitive	0.7471	0.7711	0.7589

Table 3 presents the macro precision, recall and the F1 score for the methods. The precision and recall are calculated as above, but for each article individually, and then averaged to obtain the macro precision and recall. The harmonic means of these two values for each method are the respective Macro F1 scores.

**Table 3:** Performance of stage-ethnicity extraction (macro average)

Method	Precision	Recall	F1 Score
Baseline	0.5972	1.0000	0.7478
Cost-insensitive	0.7408	0.7943	0.7666
Cost-sensitive	0.7893	0.8757	0.8302

The results in Tables 2 and 3 indicate that the cost-sensitive approach is able to significantly outperform the similar but cost-insensitive approach, which performs only close to a brute-force baseline. Not only is the cost-sensitive approach able to achieve a much higher degree of recall, but the improvement is accompanied by an increase in overall precision as well.

As the recall gets closer to the limit, the results also indicate that further improvements to the method will be gained by focusing not on extracting relevant ethnicity groups, but on eliminating the ones that are irrelevant to the article.

### Discussion of the results of task 2

The results show that a cost-sensitive committee learning approach reliably outperforms a similar, cost-insensitive approach. This holds true even when the additional committee members are weak classifiers that encode real-world domain knowledge and patterns as rules, which can compensate to some extent for the lack of data, as it is not presumable that all patterns are present in the data in significant quantity as to be learned by a model.

Some of the challenges faced in the task of extracting ethnicity groups of sample populations from the Catalog of GWAS are described below.

- *Entity normalization*: There are various ways of representing the same entity, and it is necessary to normalize these representations to a single representative entity. However, there exist degrees of difficulty with respect to normalization; for example, it is relatively easy to equate “African American” to “African-American”, but much harder to equate the two represntations with “American citizen of African origin”.
- *Studies with several target entities to extract*: Many GWAS in the U.S. use a highly ethnically diversified study sample with, for example, “52 % Caucasian, 24 % Latino, 11 % African, 9 % Eastern Asian and 4 % Indigenous Americans”, and studies may also divide into more than two stages. How to flexibly identify and deal with these situations is challenging.
- *Varying concept granularity*: Mentions of ethnicity terms might not be correctly tagged in an article as the authors may report ethnicities in specific terms such as names of tribes and indigenous people, etc., which may not map perfectly to a top-level ethnicity group. This introduces ambiguity which can be an issue for ethnicity background identification.
- *Inadequate reporting of ethnicity data*: Often, the importance of a study to a specific population or application only becomes apparent after the article is published. Hence, the text in the article may never refer to the specific ethnicity group that their experimental sample was drawn from, but simply describe it in terms of the city, state or region, or even the hospital that the population was recruited at. This is doubly challenging as it requires an indefinite expansion of the dictionary of ethnicity-related terms, and also a standardized mapping from each such term to a top-level ethnicity group.

## Conclusions

The large number of curated biomedical databases available in the public domain provides an unprecedented opportunity to train NLP systems to comprehend biomedical publications. In this paper, we present an approach to take advantage of this opportunity. The approach applied methods from learning from noisy-label and committee classifiers to assign costs to train cost-sensitive classifiers. We tested our approach for two challenging biomedical information extraction tasks. The results show that our approach is effective and outperforms alternative approaches. We will continue to investigate if it is possible to define standard passage extractors and weak learners applicable to extract biomedical entities, attributes and relations of common interest to enable rapid development and portability between domains for biomedical literature mining.

## Declarations

Research reported in this manuscript and the publication costs for this manuscript were supported by the National Human Genome Research Institute (NHGRI) of the National Institutes of Health under award number U01HG006894. The content is solely the responsibility of the authors and does not necessarily represent the official views of the National Institutes of Health.

## Additional file

Additional file 1
**Complete list of the features used for Task 2.** (CSV 1.16 kb)
