# Erratum to: Weakly supervised learning of biomedical information extraction from curated data

**DOI:** 10.1186/s12859-016-0925-9

**Published:** 2016-02-11

**Authors:** Suvir Jain, Kashyap R. Tumkur, Tsung-Ting Kuo, Shitij Bhargava, Gordon Lin, Chun-Nan Hsu

**Affiliations:** 1 Department of Computer Science and Engineering, Jacobs School of Engineering, University of California, 9500 Gilman Drive, La Jolla, San Diego, 92093 USA; 2 Department of Biomedical Informatics, School of Medicine, University of California, 9500 Gilman Drive, La Jolla, San Diego, 92093 USA

After publication of this article [1] it has been found that the name of the second author’s last name had been accidentally left out. The correct name is Kashyap R. Tumkur which has been herewith corrected in this erratum.
